# Dietary sources of free, added, and total sugars in Swedish adolescents

**DOI:** 10.1007/s00394-024-03568-8

**Published:** 2024-12-30

**Authors:** Julia Wanselius, Anna Karin Lindroos, Lotta Moraeus, Emma Patterson, Christina Berg, Christel Larsson

**Affiliations:** 1 Department of Food and Nutrition, and Sport Science, University of Gothenburg, Gothenburg, Sweden; 2 Division for Risk and Benefit Assessment, Swedish Food Agency, Uppsala, Sweden; 3 Department of Food Studies, Nutrition and Dietetics, Uppsala University, Uppsala, Sweden

**Keywords:** Free sugars, Added sugars, Sugary foods and beverages, Food sources, Adolescents, Sugar sweetened beverages

## Abstract

**Purpose:**

Swedish adolescents’ free and added sugars intake exceeds recommended levels. This poses potential health problems; however, little is known about dietary sources within the Swedish population. This study investigated dietary sources of sugars among Swedish adolescents, as well as timing and location of free sugars intake.

**Methods:**

A nationally representative sample of 3099 adolescents in school years 5, 8 and 11 (ages around 12, 15 and 18) was derived from the Riksmaten Adolescents 2016-17 cross-sectional survey. Dietary intake was self-reported over two non-consecutive days of retrospective registration. Various food categories’ contribution to sugars intake were evaluated in relative and absolute terms. To analyse differences between subsamples in consumption, non-parametric tests and logistic regression analyses were performed.

**Results:**

Sugar sweetened beverages (SSBs) were the biggest source of free (30%) and added sugars (34%) within the population, contributing with 4.4% of total energy intake among consumers. SSBs were particularly consumed by boys, adolescents to parents with lower education levels, and those residing in smaller cities/rural areas. Other food categories contributing substantially to free sugars intake were sweets and chocolates (20%), sweet bakery products and desserts (11%), fruit juices (11%), and sweetened dairy products (9%). Intakes of free sugars were higher during weekends, mostly consumed outside of main meals, predominantly within the home environment.

**Conclusion:**

The majority of free and added sugars consumed by Swedish adolescents comes from nutrient-poor food sources. SSB intake is highly associated with free and added sugars intake and is the primary source of sugars in the adolescent diet.

## Introduction

The consumption of sugars, in particular free sugars and added sugars, has become a major public health concern due to its association with various adverse health outcomes. The relationship between high sugars intake and dental caries is widely recognized [1], while there are other associated health risks suggested, including obesity, diabetes type 2 and cardiovascular diseases [2], where specifically sugar sweetened beverages (SSBs) are strongly associated with aforementioned negative health effects [2]. Also, exaggerated sugars intake provides excess energy with low nutritional value. As a result of these concerns, authorities recommend limiting sugars intake to support overall health. There are various terms used to differentiate ‘unhealthy’ sugars from naturally occurring sugars, frequently focusing on refined sugars added to foods during processing or preparation. For some time, recommendations have primarily focused on limiting the intake of ‘added sugars’ [3–5], while recently moving the focus to limiting intake of ‘free sugars’ [2, 6–8], typically to a maximum of 10% of the total energy intake per day. Free sugars are all added sugars plus the inclusion of sugars from some other natural sources, but there are variations in definitions among different stakeholders. The main and persistent difference between free and added sugars across various definitions is the inclusion of fruit juice within the definition of free sugars [2, 6, 8]. As sugars conforming to free and added sugars definitions cannot be differentiated from naturally occurring sugars by their chemical composition (hence they are all merely mono- and disaccharides), estimates of population intakes of sugars relies on different estimation methods and different definitions or proxies for free or added sugars. Due to the difficulties in intake assessment of free and added sugars, intake data among adolescents are relatively limited, especially regarding free sugars, with few countries globally providing data on sugars intake [9].

In Sweden, almost 40% of adolescents’ total energy intake comes from discretionary foods and drinks [10], defined as foods contributing with energy from saturated fat, sugars and/or alcohol, but that are of low nutritional value to the diet [11]. Estimates of free and added sugars intake among Swedish adolescents indicate that recent intake levels are above current recommendations [12], with median habitual free sugars intake of 12% of total energy (E%). From which dietary sources free and added sugars origin within the adolescent diet has not been thoroughly explored in Sweden. Nor has disparities in sugars consumption with regards to sociodemographic factors or when and where sugars are consumed been reported. These results are of importance given their potential to enable identification of intervention strategies and facilitate the customization of public health messages to align with sugars recommendations. Recently, the Nordic nutrition recommendations have shifted from focusing on added sugars only to include free sugars [8], with subsequent new dietary recommendations in Sweden emphasizing restrictions in free sugars intake [8]. This change highlights the need to examine free sugars intake, while also exploring food sources under both sugar definition distinctions, as many countries only report added sugars.

This study aimed to describe and identify contributing food sources to free, added, and total sugars intake among Swedish adolescents, explore differences in consumption of sources of free sugars between sociodemographic groups, and investigate when and where free sugars are consumed.

## Methods

### Study design and population

National representative data of Swedish adolescents in school years 5, 8 and 11 (mean ages 12, 15 and 18 years respectively) were used for this study, derived from the Riksmaten Adolescents 2016-17 survey. The survey is a cross-sectional dietary survey in Sweden, carried out by the Swedish Food Agency. Data were collected during fall 2016 and spring 2017 to capture seasonal variability. Study design and methods have previously been described [13]. In summary, the study was conducted class-wise in schools randomly selected from the national school register with sampling based on school size, geographic area, and municipality characteristics. In participating schools, 1–2 classes were recruited. Instructions on how to report dietary intake were given from trained staff from the Swedish Food Agency who visited all participating classes and measured participants’ height and weight with standardized equipment. The participants recorded their food intake in a web-based system, and both the participants and their legal guardians completed questionnaires on background data. Out of 5145 pupils invited, 3099 participated with complete dietary data from two days of retrospective food registration were included in the present study. Participants were representative to the Swedish population in terms of socioeconomic background and school organization, and schools covered geographical areas across Sweden with all types of municipalities represented [13]. Each school municipality was categorized according to the Swedish Association of Local Authorities and Regions [14], grouping them into ‘Large cities and municipalities near large cities’ (Large cities), ‘Medium-sized towns and municipalities near medium-sized towns’ (Medium-sized towns), and ‘Smaller towns/urban areas and rural municipalities’ (Smaller towns/rural areas).

### Dietary intake assessment

Dietary intake was assessed on two non-consecutive days using the web-based “RiksmatenFlexDiet”, a biomarker-validated dietary assessment method comparable to the 24-h recall method [15]. With RiksmatenFlexDiet, the participants recalled what they had eaten and drunk the day before registration. The first recall took place in school during a visit from the Swedish Food Agency staff. The second recall day was randomly assigned to each participant within 2–7 subsequent days, to ensure a representative spread between weekdays (Mondays to Thursdays) and weekends (Friday to Sunday) and could be recalled at any location [13]. The participants registered their intake by selecting foods consumed from a food list adapted for the study population. They specified intake amounts either as standard portions sizes, pieces, in household measurements or through portion pictures. The participants also reported meal type out of six alternatives (‘breakfast’, ‘lunch’, ‘dinner/supper’, ‘snack’, ‘other eating occasion’, or ‘drink only’) and eating location out of nine alternatives (‘home’, ‘school’, ‘restaurant/bar/café’, ‘event e.g., cinema/theatre/sports’, ‘someone else’s home’, ‘street food/convenience stores’, ‘work’, ‘while traveling’, or ‘other place’). Before submission, probing questions about easily forgotten foods were asked, and lastly the participants reviewed their intake amounts and eating occasions. One day’s completion time was about 15–30 min, depending on the age of the participant [15].

The list of foods items used in “RiksmatenFlexDiet” comprised 778 foods and drinks linked to the Swedish Food Agency’s food composition database, version Riksmaten adolescents 2016–2017, allowing for direct calculation of total sugars (total mono- and disaccharides) and energy intake. Contents of added and free sugars were calculated from total sugars for all food items according to a previously described systematic method [12], giving each food item an individual sugars value. Added sugars were defined in accordance with the Nordic Nutrition Recommendations and European Food Safety Authority [2, 8] as being sugars from all foods where refined sugars have been added during cooking or manufacturing, not including honey or unsweetened juices. Free sugars were defined according to WHO’s definition [6]; sugars from all food items containing added sugars, as well as sugars naturally present in honey, syrups, fruit juice and fruit juice concentrate.

### Food categorization

Foods were categorized based on similarities in food composition and culinary use. In total 14 food categories were created (Table 1). Foods with low contribution to overall sugars intake or with few consumption occasions that did not fit in any of the established categories were categorized as ‘Miscellaneous’.

**Table 1:** Food category description

Food category	Description
Breads	All types of plain breads and rolls, including yeast breads, crisp breads, and tortillas.
Breakfast cereals and porridge	Breakfast cereals, mueslis, porridge, and gruel.
Dairy products (sweetened)	Sweetened yoghurt, sour milk, fresh cheese, condensed milk, milk drinks, and plant-based dairy alternatives.
Dairy products (unsweetened)	Unsweetened milk, yoghurt, sour milk, fresh cheese, cured cheese, butter, butter blends, cream, crème fraiche, sour cream, and plant-based dairy alternatives.
Fruits and vegetables	All types of fruits and vegetables, including berries, legumes, and mushrooms. Fresh, frozen, dried, cooked, canned, or candied.
Juices (100% fruits and/or vegetables)	Juices from 100% fruits and/or vegetables.
Miscellaneous	Alcoholic beverages: beer, wine, hard cider, spirits, and liqueurs. Nutritional products: energy and protein bars, meal replacements, and protein supplements. Nuts: all sorts of whole nuts (natural, salted, spiced, or candied). Snack foods: crisps and popcorn.
Mixed dishes	Mixed dishes, home cooked and ready-made foods, including whole or products from meat, poultry, fish, seafood, eggs, plant-based meat alternatives, oils, margarines, potatoes, pasta, rice, and pseudocereals. Cold cuts. Mixed dishes and products including vegetables and legumes.
Sauces and condiments	Sauces, dressings, condiments (e.g. ketchup, mustard, mayonnaise, and sweet chili sauce), and dips for savoury foods.
Sugar sweetened beverages	Soda, cordial, energy drinks, and sugar sweetened juice drinks.
Sugars, syrups, and honey	Sugars, syrups, and honey.
Sweet bakery products and desserts	Sweet pastries, cakes, cookies, ice cream, desserts (e.g. chocolate pudding, pannacotta, tiramisu), sweet fruit soup, fruit cream, and dessert toppings (e.g. chocolate and caramel sauce).
Sweet spreads	Jams, marmalades, jellies, apple sauce, and sweet nut and cocoa spread.
Sweets and chocolates	Sweets and chocolates.

### Data analysis

The contribution to sugars intake from different food categories was calculated across all participants, i.e. proportional to total intake of sugars, and per consumer. For the proportional calculations, all participant’s two-day records were summarized and divided into the defined food categories, expressed as percent contribution to total intake (%). For the per consumer analyses, only participants who reported food items within the respective food categories were included. Food category intake per consumer is expressed as percent of total energy intake (E%) to adjust for differences in energy intake/requirement.

To address discrepancies in participant counts across different days of the week, each day was assigned a weight of 1/7 to ensure equal participant contribution from all days. This was done for individual days and not for dividing weekday/weekend as it was accounted for during sampling. When individuals had reported intake for two weekdays or two weekend days (true in a few instances), their respective individual mean values were used when comparing intakes from weekdays versus weekends.

As intake distributions are skewed, per consumer intakes are expressed in medians (p50) and interquartile ranges (p25; p75). Mann-Whitney U test was used to compare the distribution between sexes. Kruskal-Wallis test was used to compare population medians across school years. Dunns test was computed as a post hoc analysis for significant variables to conduct pairwise comparisons between school years. To explore if certain sociodemographic factors were associated with higher odds of being a consumer of the most important food categories of free sugars, logistic regression analyses were computed. The analyses were carried out stepwise, first on single variables and thereafter with models including the statistically significant variables. Independent variables in the models were sex, school year, BMI status according International Obesity Task Force cutoffs [16], highest attained parental education level and school municipality size according to Swedish Association of Local Authorities and Region’s classification [14]. To further explore the credibility of the results, an additional analysis was conducted wherein only plausible energy reporters were included, given the well-documented bias of misreporting within dietary intake assessment. Plausible energy reporters were determined by comparing energy intake captured by RiksmatenFlexDiet with total energy expenditure following the methods of Goldberg and Black [17, 18], utilizing data derived from accelerometers, body weight, and height [19]. The additional analysis was carried out on the subset of plausible energy reporters with full models. Statistical analyses were performed using Stata version 18.0. (StataCorp. 2023. Stata Statistical Software: Release 18. College Station, TX: StataCorp LLC).

## Results

### Study population

The sample included 3099 adolescents evenly distributed between the three school years, with girls comprising 55%. Characteristics and daily intakes of energy and sugars of the study sample is presented in Table 2. Within the total sample, 72% had normal weight, 61% had parents with and education level > 12 years, and the majority lived in medium sized towns (42%) as opposed to large cities (29%) and smaller towns/rural areas (29%). Median intakes of free, added, and total sugars among all adolescents were 11 E%, 9.1 E%, and 19 E%, respectively.

**Table 2:** Participant characteristics and daily intakes of energy and sugars

		School year 5		School year 8		School year 11	
		Girls (*n* = 559)	Boys (*n* = 490)	Girls (*n* = 574)	Boys (*n* = 476)	Girls (*n* = 577)	Boys (*n* = 423)
**BMI status (%)** ^**a**^							
	Underweight	8.7	6.7	7.0	7.4	5.2	5.7
	Normal weight	69	72	75	76	73	68
	Overweight	18	19	15	13	18	19
	Obese	4.4	3.1	2.3	4.2	4.5	7.4
**Parental education level (%)** ^**b**^							
	≤ 12 years	35	39	35	39	44	42
	> 12 years	65	61	65	61	56	58
**School municipality size (%)**							
	Large cities	37	31	27	28	24	27
	Medium sized towns	32	40	56	52	36	38
	Smaller towns/rural areas	31	29	17	20	40	35
**Daily intakes of energy and sugars (median (p25; p75))**							
	Energy (MJ/d)	7.5 (6.0; 9.2)	7.9 (6.4; 10)	7.8 (6.3; 9.6)	10 (7.7; 13)	7.9 (6.3; 9.8)	10 (8.2; 13)
	Free sugars (g/d)	49 (27; 74)	44 (24; 75)	49 (28; 75)	60 (33; 95)	52 (30; 86)	59 (35; 96)
	Added sugars (g/d)	38 (19; 64)	37 (18; 65)	41 (22; 66)	52 (27; 83)	44 (25; 74)	49 (25; 82)
	Total sugars (g/d)	88 (63; 120)	90 (63; 120)	91 (61; 120)	110 (80; 160)	91 (62; 130)	100 (74; 150)
	Free sugars (E%)	11 (6.9; 16)	10 (5.6; 15)	11 (7.0; 16)	11 (6.3; 16)	11 (7.3; 16)	9.8 (6.0; 15)
	Added sugars (E%)	9.3 (4.8; 14)	8.2 (4.4; 13)	9.5 (5.4; 14)	8.8 (5.0; 14)	9.7 (5.9; 14)	8.4 (4.6; 13)
	Total sugars (E%)	20 (16; 25)	19 (15; 24)	20 (16; 25)	19 (15; 24)	19 (15; 24)	17 (14; 22)

Mean age school year 5, 8, and 11: 12, 15, and 18 years old

^a^ The number of missing values varied between 0.2 and 2.2% across categories

^b^ The number of missing values varied between 3.8 and 10% across categories

### Food sources of sugars in adolescents’ diet

**Fig. 1:**
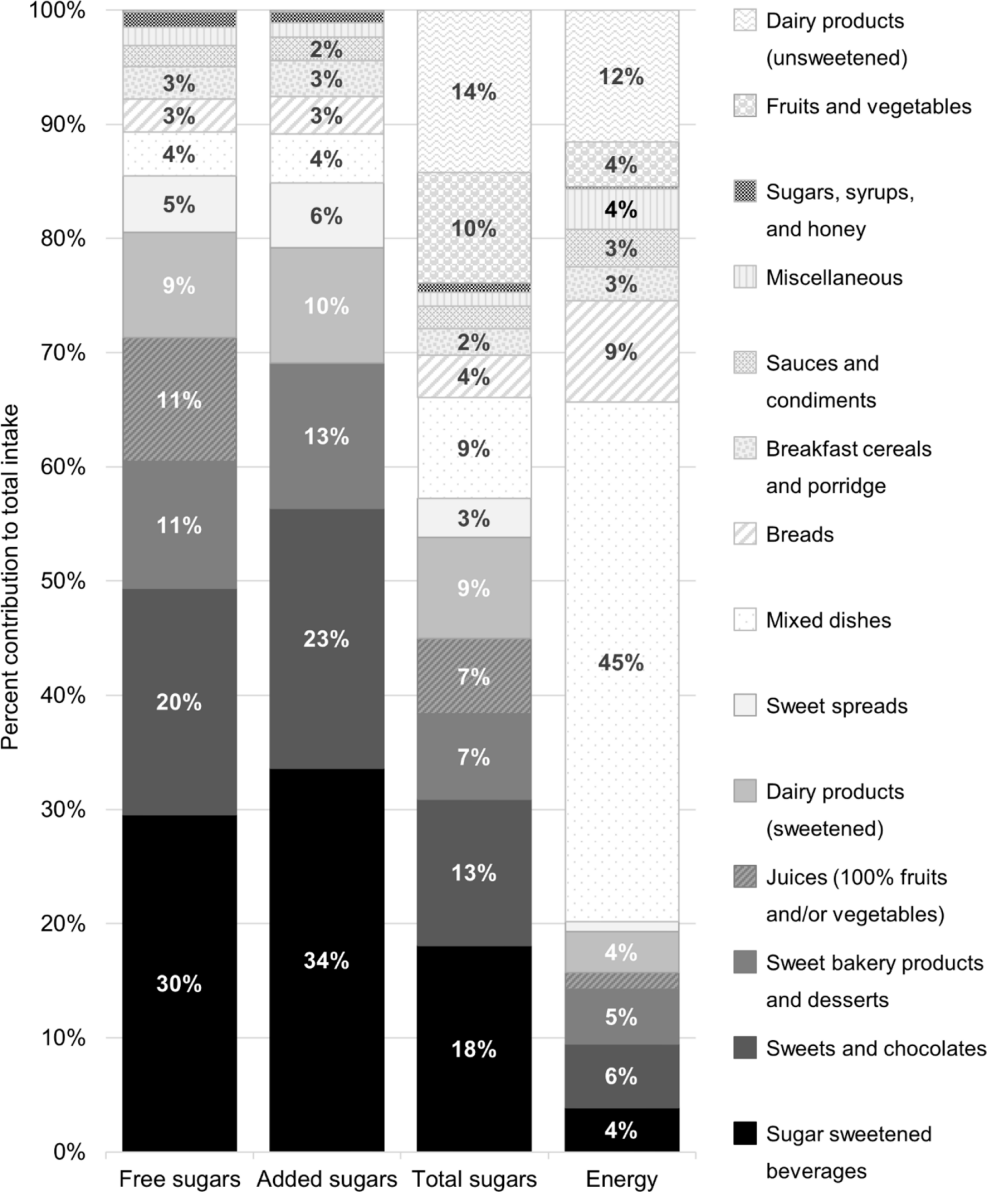
Food source contribution (%) to total intake of free sugars, added sugars, total sugars, and total energy intake in Swedish adolescents. Food categories are ranked according to contribution to free sugars intake. Food category ‘Miscellaneous’ comprises alcoholic beverages, nutritional products, nuts, and snack foods (e.g. crisps, popcorn). Food category ‘Mixed dishes’ comprises foods commonly included in main meals (e.g. pasta, rice, potatoes, meats, fish). Numbers are displayed when food categories are contributing with > 2% to total intake

The contribution of each food category to total intake of free, added, and total sugars as well as to total energy intake in adolescents’ diets is shown in Fig. 1. The top five food categories contributing to free sugars intake were the same among adolescents in all school years and between sexes (SSBs, sweets and chocolates, sweet bakery products and desserts, juices, and sweetened dairy products), adding up to 81%. SSBs were the leading contributor to overall intakes of free, added, and total sugars, with contributions of 30%, 34% and 18% respectively. SSBs contributed the most to free sugars intake across all participant groups, except for girls in school year 8, where sweets and chocolates contributed more. The oldest boys had the highest intake of SSBs, which contributed with 45% of their free sugars intake. Within SSBs, the primary source of free sugars intake was sodas (20% contribution to free sugars). SSBs together with sweets and chocolates accounted for half of the intake of free sugars and 57% of the contribution to added sugars. Within the food category sweet bakery products and desserts, primary contributors to free sugars intake were cakes, cookies, and sweet buns (7.8%). These three primary food categories contributing to sugars intake provided 15% of the total energy intake, primarily consisting of empty calories. Within the food category of sweetened dairy products, the main contributors to free sugars intake were milk drinks (4.8%), with hot chocolate accounting for a substantial share (3.1%), and flavoured yoghurt (3.7%). The main difference in contribution between free and added sugars was in juices (basically fruit juice), which contributed 11% to free sugars intake and, by definition, not at all to added sugars.

Table 3 presents per consumer free sugars intake from the food categories contributing most to total energy intake. Among consuming participants, highest median contribution was from SSBs (4.4 E%), where older adolescents had higher intakes of free sugars from SSBs than younger. SSBs, with a substantial impact on free sugars intake, were also the food category that most adolescents reported to consume (61%). Juices ranked second in contribution to free sugars among all consumers (3.1 E%), with younger adolescents demonstrating higher E% intakes compared to older. Median intakes of sweets and chocolates were 3.1 E% among all consumers, where participants in school years 5 and 8 had higher E% intakes than participants in school year 11. Among consumers, girls demonstrated higher E% free sugars from sweet bakery products and desserts than boys. In general, boys had higher intakes of sugars, syrups, and honey than girls, although few participants reported consumption of these foods.

**Table 3:** Per consumer median intakes of free sugars as percent of total energy (E%) from food categories contributing most to free sugars

			School year 5				School year 8				School year 11					
	All (*n* = 3099)		Girls (*n* = 559)		Boys (*n* = 490)		Girls (*n* = 574)		Boys (*n* = 476)		Girls (*n* = 577)		Boys (*n* = 423)			
Food category	Cons. %	Median E% (p25; p75)	Cons. %	Median E% (p25; p75)	Cons. %	Median E% (p25; p75)	Cons. %	Median E% (p25; p75)	Cons. %	Median E% (p25; p75)	Cons. %	Median E% (p25; p75)	Cons. %	Median E% (p25; p75)	p sex^†^	p school year^‡^
Sugar sweetened beverages	61	4.4 (2.6; 7.6)	61	3.6 (2.2; 6.1)	61	4.0 (2.6; 7.2)	54	4.4 (2.7; 7.1)	65	4.4 (2.7; 7.8)	61	4.9 (2.8; 8.1)	68	5.1 (2.9; 8.5)	0.055	< 0.001^abc^
Juices (100% fruits and/or vegetables)	37	3.1 (1.9; 4.6)	44	3.4 (2.1; 5.1)	30	3.5 (2.2; 5.2)	41	3.1 (1.9; 4.7)	36	2.8 (1.8; 4.3)	33	2.6 (1.8; 4.1)	34	2.7 (1.8; 4.2)	0.580	< 0.001^abc^
Sweets and chocolates	44	3.1 (1.0; 6.6)	43	3.9 (1.3; 6.6)	30	4.0 (1.3; 7.7)	60	3.1 (1.0; 6.5)	39	4.0 (1.4; 7.8)	53	2.4 (0.8; 5.8)	32	2.0 (0.5; 5.4)	0.377	< 0.001^bc^
Sweet bakery products and desserts	49	1.9 (1.1; 3.6)	54	1.8 (1.1; 3.4)	39	1.7 (0.9; 3.2)	57	2.1 (1.1; 3.9)	40	1.7 (0.9; 3.3)	58	2.3 (1.2; 3.8)	41	1.8 (1.1; 3.3)	0.005	0.116
Dairy products (sweetened)	46	1.9 (1.2; 3.2)	53	2.2 (1.4; 3.6)	43	2.1 (1.4; 3.8)	48	1.9 (1.2; 2.8)	39	1.9 (1.1; 3.4)	51	1.9 (1.2; 2.9)	41	1.6 (1.1; 2.6)	0.819	< 0.001^ab^
Sweet spreads	30	1.4 (0.8; 2.5)	34	1.4 (0.8; 2.6)	34	1.7 (0.7; 3.2)	27	1.3 (0.8; 2.2)	29	1.1 (0.7; 2.4)	29	1.4 (0.9; 2.2)	26	1.1 (0.7; 2.5)	0.748	0.069
Sugars, syrups and honey	9.8	1.3 (0.7; 2.1)	11	1.2 (0.6; 2.1)	8.0	1.5 (0.7; 2.3)	11	1.0 (0.6; 1.6)	7.1	1.6 (1.0; 3.2)	12	1.2 (0.7; 2.1)	8.0	1.5 (1.0; 2.0)	0.006	0.772
Breakfast cereals and porridge	49	0.5 (0.1; 1.0)	51	0.4 (0.1; 1.1)	53	0.5 (0.1; 1.1)	48	0.5 (0.2; 0.9)	50	0.6 (0.2; 1.2)	45	0.4 (0.1; 0.9)	45	0.4 (0.1; 0.9)	0.112	0.055

Food categories are ordered according to median intake of free sugars in all

Cons. %, percent consumers, i.e. only participants who reported consumption of foods in the food category tested

^†^ Mann-Whitney U test was used to compare the distribution between sexes

^‡^ Kruskal-Wallis test was used to compare population medians across school years. Dunns test was computed as a post hoc analysis for significant variables to conduct pairwise comparisons between school years

^a^ Differences observed between school years 5 and 8

^b^ Differences observed between school years 5 and 11

^c^ Differences observed between school years 8 and 11

**Table 4:** Top five food sources of free sugars among adolescents by quintile of free sugars E% intake

	Quintile 1		Quintile 3		Quintile 5	
	Free sugars intake < 5.7 E% (median 15 g)		Free sugars intake 9.2–12 E% (median 54 g)		Free sugars intake > 17 E% (median 112 g)	
Rank	Food category	% free sugars	Food category	% free sugars	Food category	% free sugars
1	Sugar sweetened beverages	15%	Sugar sweetened beverages	29%	Sugar sweetened beverages	34%
2	Dairy products (sweetened)	13%	Sweet bakery products and desserts	12%	Sweets and chocolates	30%
3	Mixed dishes	12%	Dairy products (sweetened)	12%	Juices (100% fruits and/or vegetables)	9.8%
4	Breads	11%	Sweets and chocolates	12%	Sweet bakery products and desserts	9.3%
5	Sweet bakery products and desserts	10%	Juices (100% fruits and/or vegetables)	12%	Dairy products (sweetened)	6.3%

E%, percent of total energy intake

In Table 4, food categories are ranked by their contribution to overall intake of free sugars by quintile of intake, showing intake quintiles 1, 3 and 5. In intake quintile 5, comprising the adolescents with the highest free sugars intakes, SSBs ranked first, sweets and chocolates ranked second, and juices ranked third. SSBs ranked the most contributing food category to free sugars intake across all intake quintiles, although the proportional contribution gradually increased with increasing quintiles, as was also apparent for sweets and chocolates. Contributions from sweet bakery products and desserts, and juices were relatively comparable across the intake quintiles, ranging between 9.3 and 14% and 9.2–13% respectively. Fewer food groups contributed substantially to free sugars intake in the higher quintiles as compared to the lower, where the top 5 contributing sources in quintile 5 totaled almost 90% of the free sugars intake. Median intakes of free sugars from food categories across the quintiles were of varying magnitudes. For SSBs, the median daily free sugars intake among consumers in the highest quintile was 40 g, 20 g in the third quintile, and 11 g in the lowest. E.g., 40 g of free sugars from sodas is equivalent to 400 ml of the beverage. Regarding sweets and chocolates, there were larger discrepancies in free sugars intakes among consumers, 43 g, 13 g, and 2.3 g per day for the three quintiles, respectively. Meanwhile, the median free sugars intakes from juices were narrower, with consumers reporting median daily intakes of 18 g, 17 g, and 11 g in the highest, middle, and lowest quintiles, respectively.

Table 5 shows the associations of sociodemographic and health related characteristics with the odds of being a consumer (i.e., to report intake of a specific food category on at least one of the reporting days) of the food categories contributing most to free sugars. Boys were more likely than girls to report SSBs, but less likely to report sweets and chocolates, sweet bakery products and desserts, and juices. Adolescents to parents with higher education level were less likely than those of lower to report SSBs and more likely to be consumers of sweet bakery products and desserts. Adolescents in smaller towns/rural areas were more likely than those in large cities to report SSBs, but they were, as well as adolescents in medium sized towns, less likely to report sweets and chocolates. Concerning BMI status, adolescents with normal weight or underweight demonstrated a higher likelihood of reporting sweets and chocolates, sweet bakery products and desserts, and juices compared to adolescents with overweight or obesity. However, when restricting the analysis to plausible energy reporters only, constituting 61% of 2232 participants with available information, no significant differences were observed between BMI statuses regarding consumption of sweets and chocolates or of sweet bakery products and desserts. For sweet bakery products and desserts, there were neither any disparities seen in consumption between parental education levels. Although, significant associations were seen in consumption of sweet bakery products and desserts, and juices between adolescents in school year 5 and year 11, where adolescents in year 11 were more likely to consume sweet bakery products and desserts than the younger (OR 1.36, 95% CI 1.03–1.78) and adolescents in year 5 were more likely to consume juices than the older (OR 1.36, 95% CI 1.03–1.80). Beyond that, the results stayed the same.

**Table 5:** Associations of sociodemographic and health related characteristics with being a consumer^1^ of the top four food categories contributing to free sugars intake. Only variables with significant associations are presented

Food category	Consumers (%)	Odds ratio	95% CI	*p*
*Sugar sweetened beverages*				
Sex				
Girls (reference)	59%	1		
Boys	64%	1.26	1.08; 1.46	0.003
Parental education level				
≤ 12 years (reference)	66%	1		
> 12 years	58%	0.75	0.64; 0.88	< 0.001
School municipality size				
Large cities (reference)	58%	1		
Medium-sized towns	60%	1.06	0.89; 1.27	ns
Smaller towns/rural areas	66%	1.37	1.12; 1.68	0.002
*Sweets and chocolates*				
Sex				
Girls (reference)	52%	1		
Boys	34%	0.49	0.42; 0.57	< 0.001
School year				
Year 5 (reference)	37%	1		
Year 8	50%	1.72	1.43; 2.07	< 0.001
Year 11	44%	1.39	1.15; 1.68	0.001
BMI status				
Normal weight or underweight (reference)	45%	1		
Overweight including obese	39%	0.77	0.64; 0.94	0.008
School municipality size				
Large cities (reference)	48%	1		
Medium-sized towns	43%	0.78	0.65; 0.93	0.007
Smaller towns/rural areas	40%	0.77	0.63; 0.95	0.013
*Sweet bakery products and desserts*				
Sex				
Girls (reference)	56%	1		
Boys	40%	0.54	0.46; 0.63	< 0.001
Parental education level				
≤ 12 years (reference)	46%	1		
> 12 years	52%	1.25	1.07; 1.45	0.005
BMI status				
Normal weight or underweight(reference)	51%	1		
Overweight including obese	43%	0.73	0.61; 0.88	0.001
*Juices (100% fruits and/or vegetables)*				
Sex				
Girls (reference)	39%	1		
Boys	33%	0.79	0.67; 0.92	0.002
BMI status				
Normal weight or underweight (reference)	38%	1		
Overweight including obese	30%	0.71	0.58; 0.86	0.001

Adjusted for significant variables in the model. Full models included sex, school year, BMI status, highest attained parental education level and school municipality size

ns, not significant

^1^ Consumers are participants who reported intakes of the specific food category on at least one of the reporting days

### Time and place of sugars consumption

**Fig. 2:**
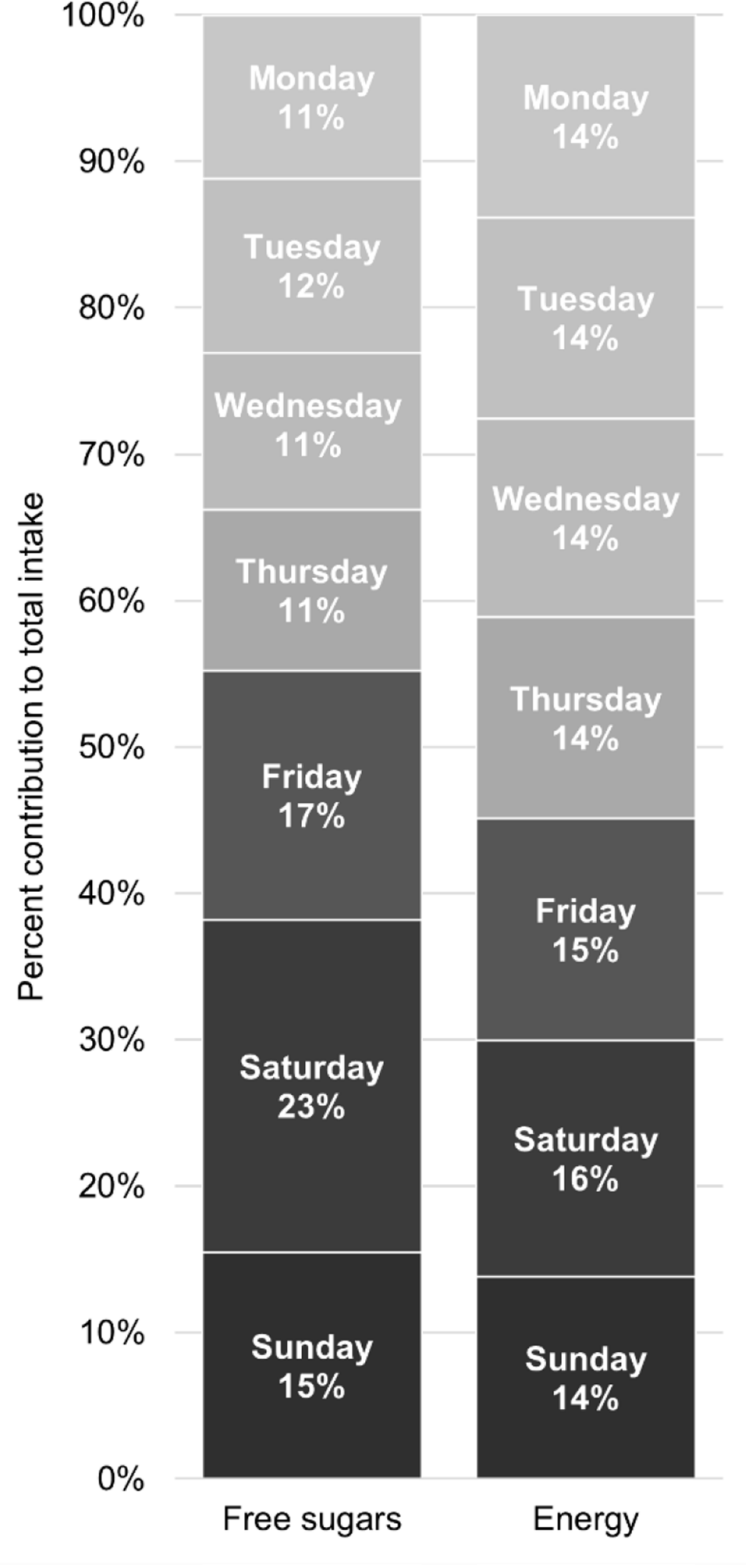
Proportional intakes (%) of free sugars and energy by day of week

Proportional intakes of free sugars and energy of adolescents by day of week are presented in Fig. 2. The day with the highest intake of free sugars was Saturday (23%), followed by Friday (17%) and Sunday (15%). The remaining days showed comparable intake proportions of free sugars. For energy intake, there was a more balanced distribution observed across days with proportional intakes from 14 to 16%, where intake was highest on Saturdays. The proportional intakes appeared similar across age groups and between sexes.

**Fig. 3:**
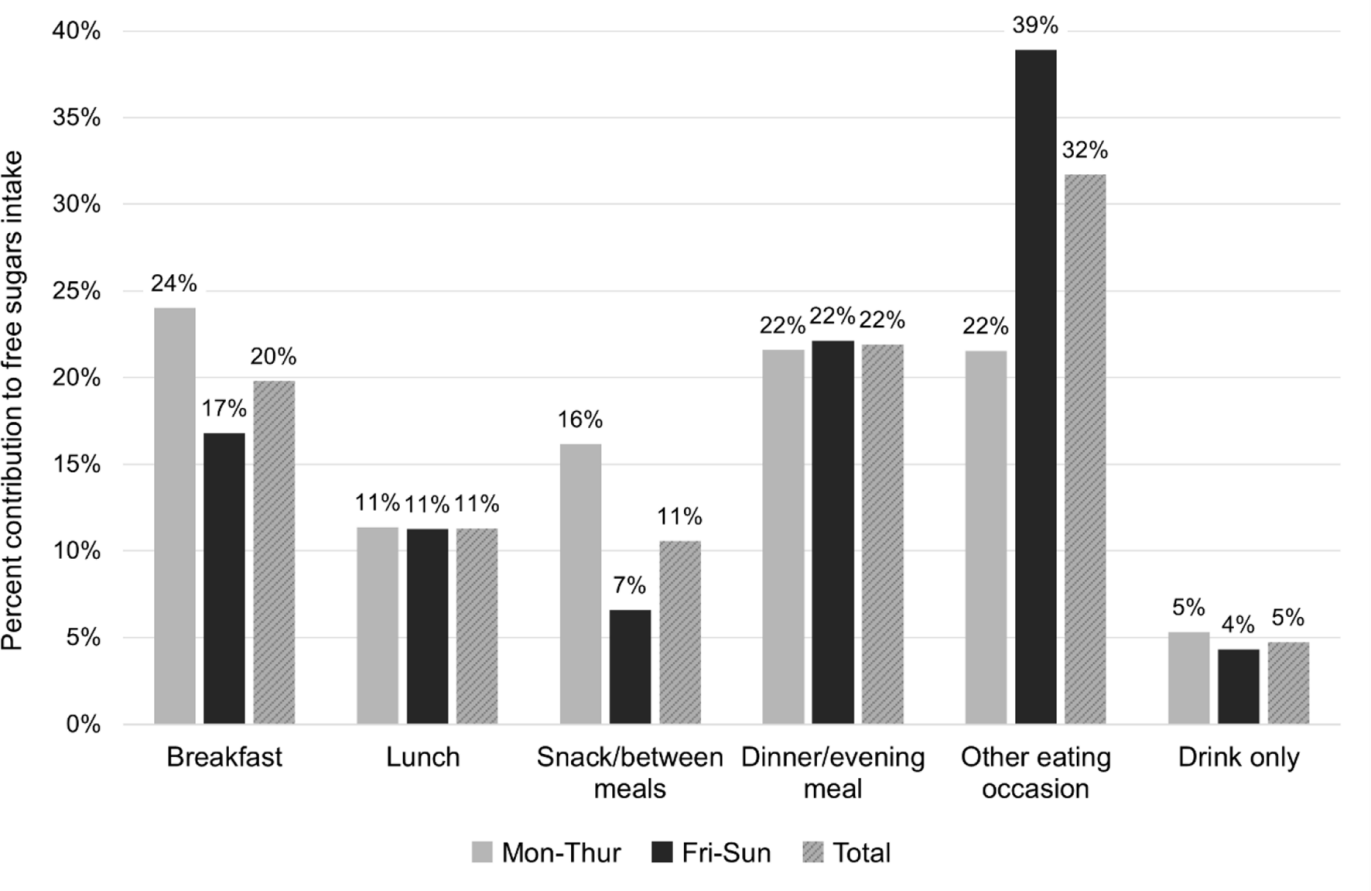
Proportional intakes (%) of free sugars by meal types during Monday to Thursday, Friday to Sunday, and total week. The meal type labels in the figure match those used in the dietary survey and participants reported meal types accordingly

Figure 3 presents the proportional intakes of free sugars by different meal types. ‘Other eating occasion’ accounted for the largest proportion of free sugars, with a notably higher contribution observed during Friday-Sunday versus on Monday-Thursday. Within this unspecified meal type, free sugars intake was predominantly derived from sweets and chocolates, comprising over half of the total intake, along with sweet bakery products and desserts, and SSBs. Intakes of these food categories constituted 93% of the free sugars intake within ‘other eating occasions’, with similar proportions observed during Monday-Thursday and Friday-Sunday. Dinner/evening meal was the second largest contributor, with equal contributions on both Monday-Thursday and Friday-Sunday. The primary source of free sugars in these meals was SSBs (56%), followed by mixed dishes (9%). Breakfasts and snacks/between meals contributed more to free sugars intake during Monday- than during Friday-Sunday. At breakfast, juices (31%) and sweetened dairy products (28%) were the main contributors to free sugars intake. At snacks/between meals, the main contributors to free sugars intake were sweet bakery products and desserts (23%), SSBs (18%), sweetened dairy products (17%), sweets and chocolates (12%) and juices (11%). The proportional intakes of free sugars reported as lunch or drink only were comparable between Monday-Thursday and Friday-Sunday. The main contributor to free sugars intake at lunch was SSBs (47%), followed mixed dishes (13%) and sweet spreads (12%). Within drinks only, the main contributor to free sugars intake was SSBs (63%), followed by miscellaneous (15%) i.e. principally free sugars from alcoholic drinks. The primary sources of free sugars remained relatively consistent across different meal types, regardless of whether it was a weekday or a weekend day. However, the proportions of the respectively food categories varied in magnitude. SSBs, along with sweet bakery products and desserts, and sweets and chocolates, generally made a larger impact to free sugars intake on Friday-Sunday than Monday-Thursday, where the contribution was more evenly distributed across various food categories.

**Fig. 4:**
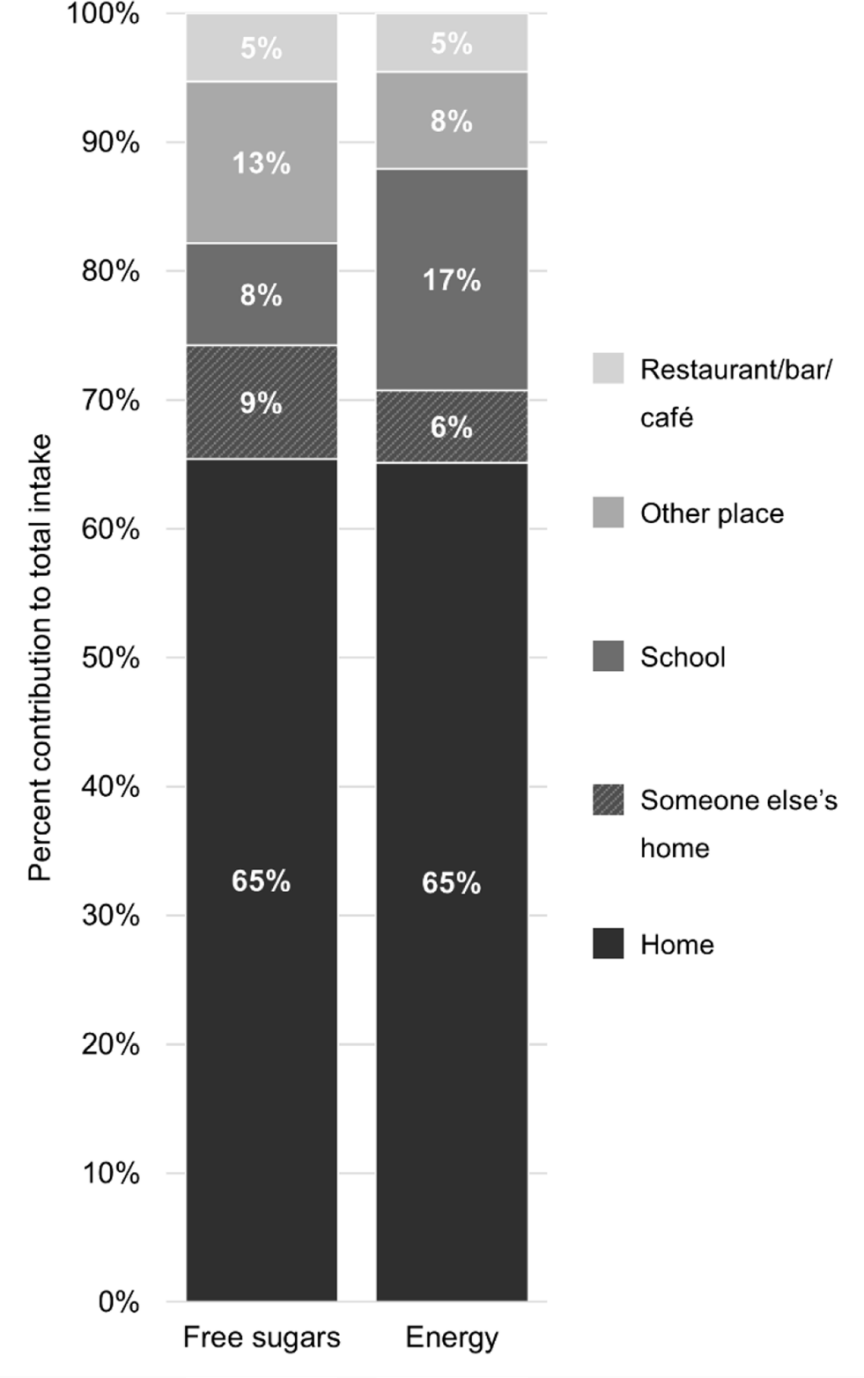
Proportional intakes (%) of adolescents’ total intake of free sugars and energy by eating location. ‘Other place’ was reported as such by the participant, and included locations: ‘event e.g., cinema/theatre/sports’, ‘street food/conven¬ience stores’, ‘while traveling’ and ‘work’

The principal eating location for consuming free sugars and energy was at home, contributing 65% to total intake respectively (Fig. 4). Following were someone else’s home and school. Regarding school, there were a noticeable discrepancy between the intake of free sugars and energy. At home, intake of free sugars was more spread across food groups (SSBs 25%, sweets and chocolates 19%, juices 14%, sweetened dairy products 12%, sweet bakery products and desserts 10%), compared to locations outside the home, where considerably more sugars came from SSBs (38%), followed by sweets and chocolates (21%), and sweet bakery products and desserts (13%).

## Discussion

This study, using national representative dietary data of adolescents, is the first to investigate dietary sources of free and added sugars in Swedish adolescents.

In coherence with findings from other studies conducted globally, where SSBs have been found to be the primary contributor to adolescents’ intake of free and added sugars [20–28] or among the top two most contributing sources [29, 30], we found that SSBs were the most contributing source to sugars intake also in this population of Swedish adolescents. In the present study, a considerable proportion of adolescents reported SSB consumption during the two registration days (61%), and of all food groups SSBs contribute most to free, added, and total sugars intake. SSBs are both the most contributing food source to the intake of free sugars in the population (30%) and constitutes the largest share of total energy intake from free sugars among consumers (4.4 E%). The intake differed between boys and girls, where boys consumed more SSBs. In fact, the oldest boys’ median intake of SSBs alone exceeded the WHO conditional recommendation [6] of a maximum of 5 E% coming from free sugars. SSBs were also the most contributing source of free sugars among high as well as low consumers of free sugars, i.e., in all intake quintiles. This is problematic as, unlike other sugar rich food items, high consumption of SSBs is well-reported as detrimental to health. Extensive evidence consistently indicates that SSB consumption is associated with health risks, including several chronic metabolic diseases [2].

In the present study, SSB consumption was not associated with overweight and obesity and the contribution to overall energy intake was 4%, which may not seem to be a substantial contribution. However, on a daily basis this might be an important addition of energy if SSBs do not substitute the energy from other foods. Moreover, SSBs together with other foods with low nutrient density contributed with 15% of energy. In coherence with other studies, we found that sweets, chocolates, sweet baked goods, and desserts were prominent sources of free and added sugars in adolescents’ diets [20–23, 26–30]. Sweets and chocolates ranked as the second contributor to both free and added sugars intakes, while sweet bakery products and desserts ranked third. These foods are more energy dense, and some of them are in addition to high in sugars also high in fat. However, in the present cross-sectional study, overweight and obese adolescents were not more likely to consume these foods compared to adolescents with normal or underweight, but rather the opposite. Inverse associations between the consumption of energy dense foods, such as baked goods and confectionery, and obesity outcomes have been reported in previous studies [31], contrasting with the commonly held assumption that such foods consistently contribute to obesity. This might to some extent be explained by underreporting, as indicated by the sensitivity analyses in the present study.

In addition to their contribution to excessive energy intake, these three food categories, including SSBs, sweets and chocolates, and sweet bakery products and desserts were contributing with 61% and 69% to the adolescent free and added sugars intake respectively, while providing low nutritional content. Fruit juices, and sweetened dairy products ranked as fourth and fifth in contributing sources to free sugars intake (20% together), providing some essential nutrients. Fruit juices also had a considerable impact on energy intake among consumers, particularly among younger adolescents. About half of the population consumed sweet bakery products and desserts, making them popular foods among the population.

For the 2022 scientific opinion on the tolerable upper intake level for dietary sugars the European Food Safety Authority (EFSA) compiled sugars intake data from the European countries, Sweden included, based on survey data from different authorities within the EU member states [32]. Using school year 5 data from the Swedish food agency’s 2003 cross sectional dietary survey about children [33], the compilation reveals SSBs being the top food source to free sugars intake among Swedish 11–12 year olds also in 2003, with 29% contribution to free sugars intake [32]. The food sources and their contributions to free sugars intake in the 2003 data are very similar to those identified in this study, with the same five main sources contributing to 88% of the free sugars intake in 2003 as compared to 81% in this study (some categorical differences in food grouping occur), indicating comparable dietary patterns over the years. The free sugars content and intake estimates are based on the same calculations here and in the EFSA compilation [12], which further reinforces this comparison.

Regarding the distribution of food sources across quintiles of free sugars intake, the intake among adolescents in the lowest quintile was sourced more evenly from various food categories, whereas fewer food groups contributed substantially with increasing intake quintiles. Particularly intakes of SSBs, and sweets and chocolates increased across the quintiles of free sugars, primarily contributing empty calories among the high consumers. A similar trend was observed in American adolescents, where sources of added sugars were examined across deciles of intakes [25]. The discrepancy in variation in food sources between adolescents with low and high free sugars intake suggest potential implications for nutritional quality. While adolescents consuming less free sugars may benefit from a varied diet potentially rich in essential nutrients, adolescents having higher free sugars intake might rely on a narrower range of foods. This raises concerns about nutrient adequacy and a potential risk of micronutrient dilution with increased intakes of free sugars, as previous studies have indicated adverse relationships between added or free sugars intake and either micronutrient intake or diet quality [34]. However, from this analysis we only know food source intake with regards to sugars intake.

Saturdays were the days with the highest consumption of free sugars. This is expected due to the Swedish “Saturday sweets” tradition, which dates back to the 1950s as a public health initiative to improve dental health by limiting sweets consumption to once a week [35]. This tradition of encouraging sweets consumption on Saturdays remains vital. In comparison with other studies reporting adolescents’ intake of sweets and chocolates as a proportion of adolescents’ free or added sugars intake, Swedish adolescents have high intakes of these foods, but with intakes more in line with adolescents from other countries within Europe [23, 26, 27, 29, 30] which are higher than those outside of Europe [20–22, 28]. Free sugars consumption among the adolescents was overall particularly high on weekends, including Fridays. Similar results were found in a sample of European children in 2007-08, where intake of both total sugars and foods and drinks rich in added sugar was higher on weekends compared to weekdays, with no significant differences in energy intake between the days [36]. These findings are in line with previous observations within the same adolescent population regarding the consumption of discretionary foods (as an indicator of foods and beverages of low nutritional value), where a similar pattern was observed with more such foods being reported during the weekend [10]. This suggests lowered nutritional quality during weekends.

A few studies outside of Europe have reported added sugars intake by eating occasion among adolescents, indicating that intakes are especially high outside of main meals [37, 38], or that snacking contribute with a substantial share of total intake [20]. Interestingly in this study, when the adolescents reported meal types of their food intake, most free sugars came from meals they did not identify as any of the normative meals including snacks (i.e. breakfast, lunch, dinner, or snack/between meals) and classified their free sugars intake as eaten during an “other eating occasion”. This may be as the Swedish word for snack typically refers to small nutritious meals, unlike the concept of snacks in many other cultures. Therefore, many sugar-rich foods consumed outside of the main meals were categorized as eaten during an “other eating occasion” rather than as a snack. However, some overlap between these two meal types occurred, as all meals were self-defined by the participants. While eating occasions outside of main meals held a large share of the free sugars intake among Swedish adolescents, it should be noted that main meals also accounted for a significant portion of overall intake.

Contrary to constant alarms within media about high contents of “hidden sugars” in everyday foods, top contributors to free and added sugars in adolescents’ diets are SSBs, and sweets and chocolates, and not foods as for example breakfast cereals, breads, condiments, or jams. One, in Sweden, commonly used and well-recognized tool for limiting sugars intake within the diet is the front-of-pack Keyhole logo [39], which guides consumers to healthier food options within different food groups. Through modelling, it has been demonstrated that by making simple exchanges in adolescents’ current diets to foods meeting the Keyhole criteria, nutrient intakes were significantly improved [40]. However, discretionary foods as SSBs, sweets, and chocolates, cannot carry the label, meaning the primary sources to free and added sugars are not addressed. Therefore, alternative strategies are necessary to effectively address adolescents’ sugars intake, and further promote healthier dietary habits.

As with any research efforts, this study has its strengths and limitations. A specific strength of this study was the application of a systematic methodology to estimate free and added sugars content in food items [12]. The food items were derived from the national food composition database, with nutrient values mainly determined through laboratory analysis, in an up-to-date food list adapted for the study population. Another strength is that the study was carried out on a representative national sample of adolescents, which, to date, stands as the only survey conducted on Swedish adolescents that is nationally representative. The representativeness allows for broader generalization to the Swedish adolescent population. However, it is important to consider the results of this study in view of the following limitations. Like in other dietary surveys, misreporting is probable in this study due to factors such as unawareness of one’s food intake and social desirability bias. Yet, it is noteworthy that a relatively high proportion of the participants in this study, compared to in other studies, had plausible energy intakes. Additionally, at group level, the level of energy reporting was found to be highly plausible [41]. As dietary intake was assessed by two 24 h recalls per participant, intakes may not reflect usual intakes on individual level. However, the primary focus of this paper is on population-level proportions, which are less sensitive to day-to-day variability in individual intake. A further strength is that data were collected across all days of the week, improving the representativeness of dietary patterns. In addition, making accurate comparisons with other studies is challenging due to discrepancies in dietary assessment methods, sugars definitions and methods to estimate sugars content of foods. However, when comparing proportions of contributing food sources, this may be less challenging, and the results of this study are within the range of previously reported findings.

## Conclusion

The majority of free and added sugars consumed by Swedish adolescents comes from dietary sources with low nutritional content. This study adds to the growing body of literature that SSBs highly contribute to and is the primary source of free and added sugars in the adolescent diet. SSBs were highly consumed by adolescents overall, particularly among boys, adolescents to parents with lower education levels, and those residing in smaller towns/rural areas. Intakes of free sugars were higher during weekends, primarily within the home environment, and mostly consumed outside of main meals. Thus, public health initiatives to reduce free or added sugars intake within the diet of Swedish adolescents should prioritize decreasing the consumption of SSBs, with a secondary focus on reducing the intake of sweets and chocolates.
